# Pretreatment Hepatitis C Virus NS5A/NS5B Resistance-Associated Substitutions in Genotype 1 Uruguayan Infected Patients

**DOI:** 10.1155/2018/2514901

**Published:** 2018-08-14

**Authors:** Fabián Aldunate, Natalia Echeverría, Daniela Chiodi, Pablo López, Adriana Sánchez-Cicerón, Alvaro Fajardo, Martín Soñora, Juan Cristina, Nelia Hernández, Pilar Moreno

**Affiliations:** ^1^Laboratorio de Virología Molecular, Centro de Investigaciones Nucleares, Facultad de Ciencias, Universidad de la República, 11400 Montevideo, Uruguay; ^2^Clínica de Gastroenterología, Hospital de Clínicas, Facultad de Medicina, Universidad de la República, 11600 Montevideo, Uruguay

## Abstract

Hepatitis C Virus (HCV) infection treatment has dramatically changed with the advent of direct-acting antiviral agents (DAAs). However, the efficacy of DAAs can be attenuated by the presence of resistance-associated substitutions (RASs) before and after treatment. Indeed, RASs detected in DAA treatment-naïve HCV-infected patients could be useful for clinical management and outcome prediction. Although the frequency of naturally occurring HCV NS5A and NS5B RASs has been addressed in many countries, there are only a few reports on their prevalence in the South American region. The aim of this study was to investigate the presence of RASs to NS5A and NS5B inhibitors in a DAA treatment naïve cohort of Uruguayan patients infected with chronic hepatitis C and compare them with reports from other South American countries. Here, we found that naturally occurring substitutions conferring resistance to NS5A and NS5B inhibitors were present in 8% and 19.2%, respectively, of treatment-naïve HCV genotype 1 infected patients. Importantly, the baseline substitutions in NS5A and NS5B herein identified differ from the studies previously reported in Brazil. Furthermore, Uruguayan strains subtype 1a clustered within all major world clades, showing that HCV variants currently circulating in this country are characterized by a remarkable genetic diversity.

## 1. Introduction

Hepatitis C Virus (HCV) infection treatment has dramatically improved thanks to the introduction of direct-acting antiviral agents (DAAs). These antivirals have significantly increased response rates (up to 98%) and greatly reduced treatment duration [1]. Currently available DAAs are classified into four categories given their molecular targets in the HCV replication cycle: (1) NS3/4A protease inhibitors (PIs) bind to the active site of the NS3/4A protease; (2) NS5A inhibitors interact with domain 1 of the NS5A dimer, although the exact mechanism of NS5A inhibition remains to be fully elucidated; (3) nucleos(t)ide analog NS5B polymerase inhibitors are incorporated into the nascent RNA chain resulting in chain termination by compromising the binding of the incoming nucleotide; (4) nonnucleoside NS5B polymerase inhibitors interact with either the thumb 1, thumb 2, palm 1, or palm 2 domain of NS5B and inhibit polymerase activity by allosteric mechanisms [2–4]. However, the extreme mutation and high replication rates of HCV, together with the immune system pressure, lead to a remarkable genetic variability that can compromise the high response rates to DAAs due to the preexistence of resistance-associated substitutions (RASs) [5, 6].

Each drug or class of DAA is characterized by specific resistance profiles. The likelihood that a DAA will select for and allow outgrowth of viral populations carrying RASs depends on the DAA's genetic barrier to resistance (the number and type of mutations needed to generate an amino acid substitution that confers resistance), the viral fitness (replicative capacity) of the resistant variant, and viral genotypes and subtypes [7, 8].

The prevalence of RASs in treatment-naïve patients has been broadly reported worldwide [9–16]. However, apart from Brazil and Argentina, this issue has not been fully addressed in other South American countries yet [9, 17–19]. The lack of information in relation to preexisting baseline RASs, added to the high cost of these new drugs, are the major limiting factors for the broad implementation of these new therapies in Uruguay as well as in other Latin American countries (low- or lower-middle income) [20].

In this study, we explored the presence of resistance variants to NS5A and NS5B inhibitors in a DAA treatment naïve cohort of Uruguayan patients chronically infected with hepatitis C. Here, we aimed to contribute to the knowledge of the circulation of HCV resistant variants in the South American region.

## 2. Materials and Methods

### 2.1. Patients and Clinical Samples

Serum samples were obtained from 31 patients with serological markers for HCV, which were recruited between 2015 and 2017 at the Gastroenterology Clinic from Hospital de Clínicas, Montevideo, Uruguay. HCV infection was confirmed by Abbott realtime HCV (Abbott Molecular Inc., Des Plaines, USA). Patients selected for this study were both chronically infected with HCV genotype 1 and DAA treatment-naïve at the time of blood extraction. Written informed consent was obtained from all patients. The studies have been performed according to the World Medical Association Declaration of Helsinki and approved by the appropriate institutional board (Hospital de Clínicas ethical committee).

### 2.2. RNA Extraction, cDNA Synthesis, and NS5A and NS5B Amplification

Viral RNA was extracted from 140 *μ*l of serum using the QIAamp Viral RNA mini kit (QIAgen, Hilden, Germany) according to the manufacturer's protocol. The viral RNA was heated at 65°C for 5 min and used as a template for a reverse transcription reaction. The reverse transcription reaction mixture contained 5 *μ*l of the RNA template, 1 *μ*l of random hexamer 100 ng/*μ*l (Invitrogen Life Technologies, Carlsbad, CA, USA), 1 *μ*l of dNTP mix (10 mM each), 4 *μ*l of 5X first-strand buffer, 2 *μ*l of 0.1 M DTT, 1 *μ*l of SuperScript II reverse transcriptase (200 U/*μ*l) (Invitrogen Life Technologies, Carlsbad, CA, USA), and 1 *μ*l (40 U/*μ*l) RNaseOUT (Invitrogen Life Technologies, Carlsbad, CA, USA). The reverse transcription was performed at 42°C for 50 min, and then the reverse transcriptase enzyme was inactivated at 70°C for 15 min. PCR amplification of NS5A and NS5B genome regions was performed using primers and conditions previously described [10]. Amplicons were purified using the Illustra GFX PCR DNA and Gel Band Purification Kit (GE Healthcare Life Science, Buckinghamshire, UK) according to the manufacturer's protocol.

### 2.3. NS5A and NS5B Sequencing

The purified product was then sequenced using the same sets of primers used for PCR amplification. Bidirectional Sanger sequencing was performed by Macrogen Korea (http://www.macrogen.com).

### 2.4. NS5A and NS5B Genotype Determination

HCV NS5A and NS5B consensus sequences obtained from Uruguayan patients were aligned with sequences from HCV representing all genotypes and main subtypes isolated in different geographic regions of the world. These sequences were obtained from Los Alamos HCV sequence database and from the NIAID Virus Pathogen Database and Analysis Resource (ViPR) [21, 22]. For strains included in these studies, see Supplementary Material Table S1. Sequences were aligned using the CLUSTAL W software [23]. Once aligned, the best evolutionary model that described our sequence data was assessed using ModelGenerator program [24]. Using the GTR + G + I model (General time reversible + gamma + invariant sites), maximum likelihood phylogenetic trees were constructed for both NS5A and NS5B using the MEGA 5.0 software [25]. For NS5A, 953 nucleotides (positions 6367 to 7319, relative to HCV 1a reference strain, H77 NC_004102) were included in the phylogenetic analysis, whereas for NS5B, only 361 nucleotides corresponding to the Okamoto region (positions 8265 to 8625, relative to strain H77 NC_004102) were included. As a measure of the robustness of each node, we employed the bootstrapping method (1000 pseudoreplicates).

For NS5A 1a Uruguayan sequences (*n* = 20), a second alignment and maximum likelihood phylogenetic tree was generated in order to analyze HCV evolutionary relationships between Uruguayan, Brazilian, and worldwide strains. For non-Uruguayan strains included in this analysis, see Supplementary Material Table S2.

### 2.5. NS5A and NS5B Sequence Analysis

In order to properly identify substitution changes in NS5A and NS5B regions from HCV strains circulating in Uruguayan patients, we generated world consensus sequences for 1a and 1b subtypes using a wide range of NS5A and NS5B sequences from HCV strains isolated worldwide. For this purpose, NS5A gene sequences corresponding to subtypes 1a (*n* = 160) and 1b (*n* = 88) were retrieved from Los Alamos HCV sequence database and from the NIAID ViPR [21, 22]. Likewise, datasets of 150 and 124 NS5B sequences were generated for subtypes 1a and 1b, respectively. Using Seqman program, implemented in DNAStar 5.01 package (DNASTAR, Madison, USA), a world consensus nucleotide sequences were generated for each gene and subtype. Each Uruguayan sequence was subsequently aligned to the corresponding reference sequences, and then in silico translated. The amino acid sequences obtained were compared in order to explore the presence of RASs as well as the presence of polymorphisms at a RAS position (RAPs) in Uruguayan HCV strains. RAPs are defined as any change from reference sequence for a specific genotype at a position associated with NS5A resistance [26].

## 3. Results

### 3.1. Genetic Variability of NS5A and NS5B Genes from HCV Strains Circulating in Uruguayan Patients

**Figure 1:**
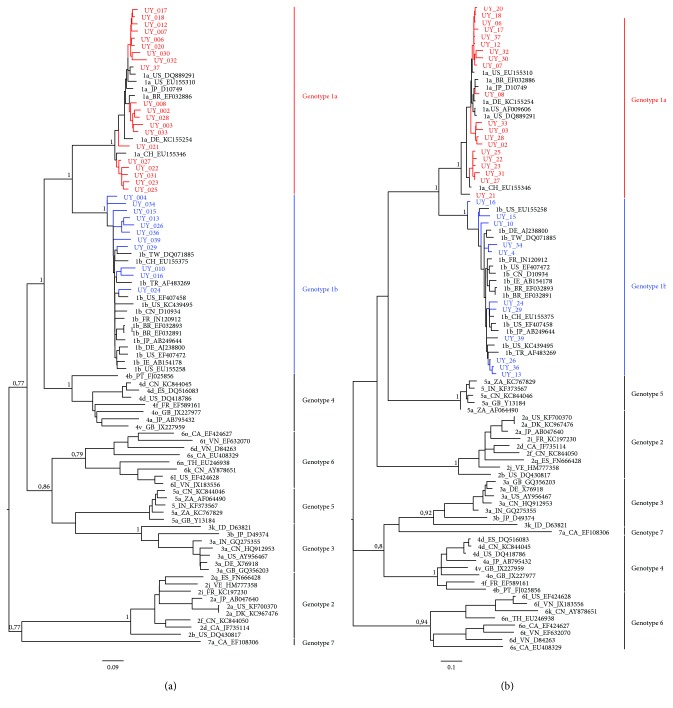
Maximum-likelihood phylogenetic trees analyses of the NS5A and NS5B genes of HCV strains circulating in Uruguay. Reference strains in the tree are shown by genotype_country_accession number. Numbers at the branches indicate bootstrap values. The bar at the bottom of the tree denotes distance. Uruguayan strains genotype 1a are shown in red and genotype 1b in blue. (a) NS5A region (953 nucleotides); (b) NS5B region (361 nucleotides).

To study the genetic variability of NS5A and NS5B regions of HCV strains circulating in Uruguayan patients, sequences of these regions (accession numbers MH070029-MH070090) were aligned with corresponding sequences from 59 HCV strains isolated elsewhere, representing all genotypes and main subtypes (for strains included in these analyses, see Supplementary Material Table S1). Therefore, maximum likelihood phylogenetic trees were constructed. The results of these studies are shown in Figure 1 (Figure 1(a): NS5A and Figure 1(b): NS5B).

All strains in the phylogenies were assigned according to their genotype, and each cluster was supported by very high bootstrap values for both analyzed regions. Strains isolated from Uruguayan patients (*n* = 31) were assigned to genotype 1, 20 of which corresponded to subtype 1a and 11 to subtype 1b. The results of NS5A (Figure 1(a)) and NS5B (Figure 1(b)) phylogenetic analyses were concordant for both genomic regions in all 31 sequences, suggesting no recombination events between these regions.

**Figure 2:**
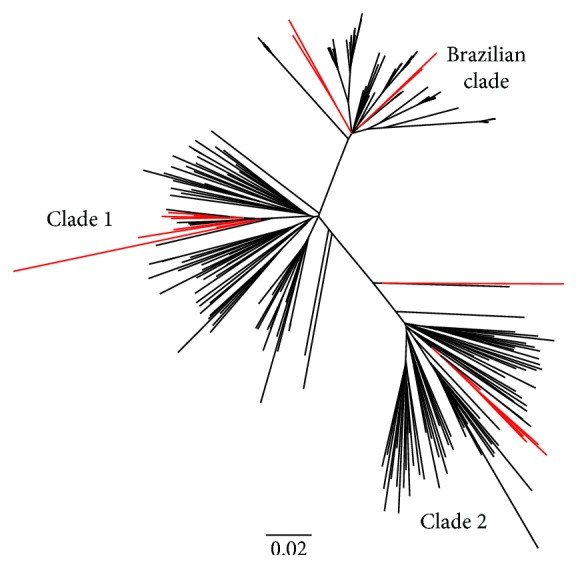
Maximum-likelihood phylogenetic tree analysis of the NS5A of HCV-1a strains circulating in Uruguay. The maximum-likelihood phylogenetic tree shows the evolutionary relationships between the Uruguayan, Brazilian, and worldwide HCV-1a isolates sequences. Uruguayan strains are shown in red. The bar at the bottom of the tree denotes distance. Sequence names have been removed for clarity.

To further analyze the evolutionary relationships between the Uruguayan strains and those circulating in Brazil and elsewhere, a second maximum likelihood phylogenetic tree of HCV-1a sequences of NS5A partial region was built (Figure 2). As was previously described, two distinct 1a clades (clades 1 and 2) were observed. Brazilian sequences clustered in a large group of related sequences inside clade 1 [9]. Whereas NS5A Uruguayan strains (in red) did not cluster in a particular clade, rather, they grouped dispersedly within all major world clades.

### 3.2. NS5A Substitution Analysis

With the purpose of studying the amino acid (AA) substitutions along the NS5A protein, Uruguayan HCV AA sequences were aligned with NS5A world consensus sequences (residues 23 to 354 relative to NS5A protein sequence). AA substitutions at positions previously found to be potentially associated with resistance to NS5A inhibitors, as well as polymorphisms at a RAS position, were identified. These results are summarized in Table 1.

RASs to NS5A inhibitors (L31M and L31V) were identified in 2 strains out of 25 (8%) fully sequenced samples. RAPs were found in 3 strains (subtype 1a): 2 exhibited the substitution H58P and 1 the substitution K24Q. Although these substitutions were not reported as resistant, some changes at these positions were previously described as RASs in subtype 1a, namely H58D and K24R [27, 28]. Finally, substitution E62D was found in one subtype 1a strain. This change is considered as a secondary substitution because, although it does not confer resistance by itself, when combined with a known RAS it does. In fact, it confers a higher level of resistance than the one achieved by the RAS alone [26]. In addition, several polymorphisms that have not been previously reported to be associated with a resistant phenotype were also detected (see Supplementary Material Table S3).

### 3.3. NS5B Substitution Analysis

In order to study substitutions along NS5B protein, Uruguayan HCV AA sequences were aligned to the NS5B world consensus sequences. Almost full-length AA sequences were obtained in 26 out of 31 analyzed strains. 23 sequences span residues 36 to 539 whereas the remaining 3 span residues 36 to 557 of NS5B protein. This issue limited our studies, since many of the described RASs are observed as of residue 553.

Importantly, RASs to NS5B inhibitors (Table 2) were observed in 5 strains out of 26 sequenced samples (19.2%). C451R was found in two isolates while A421V was found in only one. In 2 of the 3 strains for which we were able to obtain longer sequences, RASs S556G (subtype 1a) and Q556R (subtype 1b) were observed.

Finally, we found two RAPs: A421V (in 2 subtype 1b strains) and A553G (in 1 subtype 1a strain). Although A421V has been associated with resistance to beclabuvir (BCV) in patients infected with HCV subtype 1a, this resistant phenotype has not been proven in strains subtype 1b [29]. In position 553, the substitution reported as resistant was A553T [8].

As was the case for NS5A, different polymorphisms not previously associated with a resistant phenotype were also detected in NS5B (see Supplementary Material Table S4).

## 4. Discussion

The advent of DAAs therapies constitutes one of the major breakthroughs in HCV infected patients management. However, these new treatment options are far from being universally available, in particular for HCV infected patients relying on Latin American public healthcare systems. The main limiting factors for worldwide access to DAAs in our region concern the high cost, the inadequate management of public healthcare systems, the limited access of low-income or uninsured populations to healthcare providers, and the lack of accurate epidemiological information [20, 30–32]. In Uruguay, these therapies became recently available, and although some have been approved for their use by the public health authorities (Viekira pak and sofosbuvir/ledipasvir therapies), they are not currently financially covered, except in specific cases. Despite the high rates of viral response achieved with DAA-based treatments, still 1 to10% of the patients fails to eliminate infection, and in these cases, baseline and emergent resistance variants turn out to be key factors contributing to treatment failure [5, 17, 33].

Unfortunately, we are currently unable to properly assess the number of HCV infected people in Uruguay and even more to figure out the frequency and type of RASs circulating. These facts could compromise the effectiveness of these new therapies in our country.

We have previously reported that naturally occurring substitutions conferring resistance to NS3 inhibitors exist in a significant proportion of Uruguayan patients infected with HCV genotype 1, and we showed that this frequency seemed to be higher than in other South American countries (Brazil and Argentina) [34]. The present study describes the prevalence of baseline NS5A and NS5B RASs in HCV genotype 1 infected DAA-naïve patients in a Uruguayan cohort.

The presence of substitutions conferring resistance to NS5A inhibitors has been widely reported both in therapy-naïve and in relapser patients from Europe [10, 33, 35–38], USA [37, 39, 40], and Asia [41–43]. However, NS5A sequences from South America are poorly analyzed yet [9, 44]. Recent studies have revealed that the mean prevalence of NS5A genotype 1 baseline RASs to different inhibitors ranges from 6% to 16% using population sequencing or deep sequencing [27, 37, 45, 46]. Importantly, the prevalence and type of baseline NS5A RASs varies slightly by geographic regions. For instance, L31M was found in 2.2% of genotype 1a infected patients in Europe, in 4.1% of those in Oceania, and strikingly in no patient from the USA [27]. For this reason, we believe that there is a need to contribute data from our region, for which we still do not have enough information, apart from Brazil [9, 44]. The results of this study indicate the presence of DAA NS5A RASs in 2 HCV strains (8% of the patients enrolled in this study), with baseline RASs detected at position 31 (see Table 1). L31M substitution confers resistance to daclatasvir (DCV), ledipasvir (LDV), and elbasvir (EBV) in both 1a and 1b subtypes [5, 6, 8, 28, 47, 48], whereas substitution L31V does it to DCV in subtypes 1a and 1b, to LDV in subtype 1b, and to EBV in subtype 1a [5, 6, 28]. Given that both L31V and L31M are clinically relevant RASs, their detection at baseline may influence the choice of first-line treatment regimens [28].

The substitutions H58P and K24Q found in two patients are considered as resistance-associated polymorphisms (RAPs). The RASs characterized at these positions were H58D and K24G/N/R [5, 6, 27, 28, 49, 50]. The substitution H58P was found as a baseline RAP in relapsers to LDV (HARVONI prescription, https://www.gilead.com/-/media/files/pdfs/medicines/liver-disease/harvoni/harvoni_pi.pdf?la=en). However, it is sometimes regarded as a RAS [10, 51], despite conferring only 1.2 fold change in resistance in *in vitro* studies using the 1a replicon system [39].

We did not find M28T/V, Q30R/H, or Y93H substitutions as there were previously reported in Brazil and worldwide [9, 27, 44]. The amino acid substitution E62H was found in one Uruguayan patient. Although this change does not confer resistance by itself but in combination with Q30R, it generates a high resistance level to DCV [52].

The presence of baseline NS5A RASs impacts treatment outcome in some patient groups by affecting SVR rates. The detection of NS5A preexistent RASs may play a relevant role in the choice of first-line treatment regimens or in the simplification/shortening of recommended regimens, in order to bring SVR rates close to the highest achievable [27, 38, 41, 53], in particular in countries such as Uruguay, where only two different DAA-containing treatment regimens are approved for their use.

Regarding NS5B gene, global analysis (with the exception of South America [17, 19]) revealed that NS5B DAA resistance substitutions are infrequent [14]. Our study showed the presence of NS5B inhibitors RASs in 5 out of 26 analyzed HCV infected Uruguayan patients naïve to treatment (19.2%). Substitutions found in this work were A421V and S556G associated in subtype 1a with resistance to BCV and dasabuvir (DSV), respectively [8, 28, 29, 54, 55], and Q556R associated with resistance to DSV both in genotype 1a and 1b [12, 28]. Substitution C451R, observed in two Uruguayan patients, was reported previously in patients who failed to clear the infection after treatment with OBV/PTV/r + DSV ± RBV. In these cases, it appeared in combination with G558R (Trial Coral I-Cohort 2: http://www.hcv-trials.com/showStudy.asp?Study=86).

RAPs in positions 421 and 553 (A421V in two subtype 1b isolates and A553G in one subtype 1b isolate) were also found. Although A421V has been associated with resistance to BCV in patients with subtype 1a, this phenotype has not been proven in strains of subtype 1b [29]. In position 553, the substitutions reported as resistant are A553T in subtype 1a [8] and A553V in subtype 1b [54], conferring resistance to DSV.

In contrast to our results, Noble and coworkers (2016) reported the presence of V321A, A421G, M414V, Y448H, L159F, and C316N in Brazilian isolates [17], yet none of these mutations were found in this study, probably due to the diversity found between Uruguayan and Brazilian strains (Figure 2). Nevertheless, substitution A421V was found in Brazil [17], Argentina [19], and Uruguay. The RAS S282T was detected neither in Brazilian reports nor in this current work (Uruguay) [17, 18, 56]. Our findings further confirm and complement previous studies which evidenced a low prevalence of this substitution *in vivo*, probably due to its low replicative fitness [14, 18, 57]. Despite our results, it is worth mentioning that the presence of baseline NS5B RASs conferring resistance to nucleotide or nonnucleoside NS5B inhibitors has not been shown to have any impact on virologic responses thus far [53, 58].

These results show both diversity in the baseline polymorphisms found in different Latin American countries and in the evolutionary relationships of Uruguayan isolates (Figure 2). This fact could be linked not only to the isolates' geographic region and viral intrinsic characteristics but also to the genetic background of the host. It is worth mentioning that we live in a vast continent inhabited by populations with different genotypic characteristics that might, depending on the situation, require different approaches to treatment. Indeed, we have recently found that allele and genotype frequencies at *IL28B* locus of Uruguayan individuals closely resemble those of an admixed population rather than a uniformly European-descendant one [59]. Altogether, we believe that it could be important to carry out studies throughout the South American region in order to establish the prevalence of RASs in NS5A and NS5B in different countries. In fact, this will aid in understanding that not every treatment regimen might be adequate for every patient and country. The data we presented here might guide not only physicians in making therapeutic decisions but also public health authorities in approving more diverse treatment combinations. These treatment formulations would cover most of the circulating strains in our region, a region with an extremely diverse genetic background population.

## 5. Conclusion

To our knowledge, the present study revealed for the first time the presence of RASs in the NS5A and NS5B regions of HCV genotype 1 Uruguayan strains from patients who have not been previously treated with DAAs and is one of the few South American countries to report on this matter. It is currently unclear if preexisting viral variants with reduced susceptibility to DAAs are clinically relevant for the prediction of virologic treatment failure. However, individualized DAA therapy based on baseline resistance analysis may be beneficial for optimizing treatment efficacy in patients with HCV genotype 1 infection and risk factors for treatment failure. Therefore, the potential role of baseline resistance testing remains an area of critical research and clinical questions.

## Figures and Tables

**Table 1:** Amino acid substitutions in HCV NS5A protein from DAA treatment-naïve patients.

Subtype	Isolate	RAS	RAP	Resistance to DAA	Reference
1a	003		H58P	—	HARVONI prescription^∗^
1a	020		K24Q	—	[5, 49]
1a	032		H58P	—	HARVONI Prescription^∗^
1b	016	L31 V		DCV, LDV, EBV	[5, 6]
1b	039	L31 M		DCV, LDV, EBV	[5, 47, 48]

Daclatasvir (DCV), ledipasvir (LDV), elbasvir (EBV). ^∗^Harvoni prescription, https://www.gilead.com/~/media/Files/pdfs/medicines/liver-disease/harvoni/harvoni_pi.pdf.

**Table 2:** Amino acids substitutions in HCV NS5B protein from DAA treatment-naïve patients.

Subtype	Isolate	RAS	RAP	Resistance to DAA	Reference
1a	003	C451R		DSV	Trial Coral I - cohort 2^∗^
1a	020	A421V		BCV	[29]
1a	022	C451R		DSV	Trial Coral I - cohort 2^∗^
1a	037	S556G		DSV	[54, 55]
	037		A553G	—	[8]
1b	026		A421V	—	[29]
	026	Q556R		DSV	[12]
1b	036		A421V	—	[29]

Beclabuvir (BCV), dasabuvir (DSV). ^∗^Trial Coral I - cohort 2 http://www.hcv-trials.com/showStudy.asp?Study=86 (Mantry PS. AASLD, 2015, Abs. 1084).

## Data Availability

The data used to support the findings of this study are included within the article.

## Supplementary Materials

Supplementary Materials Supplementary Material Table S1: hepatitis C Virus NS5A and NS5B sequences used as representatives of each genotype to perform the phylogenetic analysis. Their corresponding genotype, country of isolation, and GenBank accession number are indicated. Supplementary Material Table S2: hepatitis C Virus NS5A subtype 1a sequences used to reveal evolutionary relationships between Uruguayan strains and others isolated elsewhere. Their corresponding country of isolation and GenBank accession number are indicated. Supplementary Material Table S3: amino acid substitutions in NS5A protein not previously associated with resistance to NS5A inhibitors. Supplementary Material Table S4: amino acid substitutions in NS5B protein not previously associated with resistance to polymerase inhibitors.
