# Supplementary material for: Pretreatment Hepatitis C Virus NS5A/NS5B Resistance-Associated Substitutions in Genotype 1 Uruguayan Infected Patients
Source: Dis Markers. 2018 Aug 14;2018:2514901. doi: 10.1155/2018/2514901 (PMC6112080; doi:10.1155/2018/2514901)
Supplement: Supplementary Materials — Supplementary Material Table S1: hepatitis C Virus NS5A and NS5B sequences used as representatives of each genotype to perform the phylogenetic analysis. Their corresponding genotype, country of isolation, and GenBank accession number are indicated. Supplementary Material Table S2: hepatitis C Virus NS5A subtype 1a sequences used to reveal evolutionary relationships between Uruguayan strains and others isolated elsewhere. Their corresponding country of isolation and GenBank accession number are indicated. Supplementary Material Table S3: amino acid substitutions in NS5A protein not previously associated with resistance to NS5A inhibitors. Supplementary Material Table S4: amino acid substitutions in NS5B protein not previously associated with resistance to polymerase inhibitors. [file 2514901.f1.doc]

**Supplementary Material Table S1.** Hepatitis C Virus NS5A & NS5B sequences used as representatives of each genotype to perform the phylogenetic analysis. Their corresponding genotype, country of isolation and GenBank accession number are indicated.

**Genotype Country of Isolation GenBank Accession Nr**

1a Brazil EF032886

United States DQ889291

United States EU155310

Japan D10749

China EU155346

Germany KC155254

1b Japan AB249644

China D10934

Taiwan DQ071885

Ireland AB154178

Germany AJ238800

China EU155375

France JN120912

Turkey AF483269

United States EF407458

United States EF407472

United States EU155258

United States KC439495

Brazil EF032891

Brazil EF032893

2a United States KF700370

Japan AB047640

Denmark KC967476

2b United States DQ430817

2d Canada JF735114

2f China KC844050

2i France KC197230

2j Venezuela HM777358

2q Spain FN666428

3a United States AY956467

India GQ275355

United Kingdom GQ356203

China HQ912953

Germany X76918

3k Indonesia D63821

3b Japan D49374

4a Japan AB795432

4b Portugal FJ025856

4d United State DQ418786

Spain DQ516083

China KC844045

4f France EF589161

4v United Kingdom JX227959

4o United Kingdom JX227977

5a South Africa AF064490

ZA KC767829

China KC844046

United Kingdom Y13184

5 - KF373567

6k China AY878651

6d Vietnam D84263

6o Canada EF424627

6l United State EF424628

Vietnam JX183556

6t Vietnam EF632070

6n Thailand EU246938

6s Canada EU408329

7a Canada EF108306

**Supplementary Material Table S2.** Hepatitis C Virus NS5A subtype 1a sequences used to reveal evolutionary relationship between Uruguayan strains and others isolated elsewhere. Their corresponding country of isolation and GenBank accession number are indicated.

**Country of Isolation GenBank Accession Nr**

| Australia | FJ932275 | FJ932344 | FJ932287 | FJ932356 |
| --- | --- | --- | --- | --- |
|  | FJ932278 | FJ932346 | FJ932292 | FJ932358 |
|  | FJ932280 | FJ932349 | FJ932293 |  |
|  |  |  |  |  |
| Brazil | EU309615 | EU309595 | EU309654 | EU309665 |
|  | EU309622 | EU309588 | EU309645 | EU309663 |
|  | EU309628 | EU309591 | EU309609 | EU309661 |
|  | EU309616 | EU309599 | EU309601 | EU309672 |
|  | EU309619 | EU309590 | EU309606 | EU309671 |
|  | EU309617 | EU309597 | EU309605 | EU309668 |
|  | EU309624 | EU309596 | EU309611 | EU309670 |
|  | EU309626 | EU309650 | EU309608 | KJ747874 |
|  | EU309623 | EU309644 | EU309604 | KJ747875 |
|  | EU309618 | EU309649 | EU309614 | KJ747852 |
|  | EU309620 | EU309658 | EU309610 | KJ747885 |
|  | EU309625 | EU309646 | EU309600 | KJ747886 |
|  | EU309627 | EU309648 | EU309612 | KJ747888 |
|  | EU309631 | EU309652 | EU309602 | KJ747889 |
|  | EU309633 | EU309656 | EU309613 | KJ747890 |
|  | EU309641 | EU309653 | EU309607 | KJ747891 |
|  | EU309637 | EU309651 | EU309662 | KJ747895 |
|  | EU309643 | EU309647 | EU309673 | KJ747878 |
|  | EU309639 | EU309655 | EU309660 | GQ848736 |
|  | EU309636 | EU309657 | EU309666 | KJ747892 |
|  | EU309632 | EU309587 | EU309592 | EU309593 |
|  | EU309629 | EU309589 | EU309598 | EU309594 |
|  | EU309630 | EU309635 | EU309642 | EU309586 |
|  | EU309640 |  |  |  |
|  |  |  |  |  |
| Canada | KX621485 |  |  |  |
|  |  |  |  |  |
| Germany | EU256107 | EU687194 | EU862832 | JQ914273 |
|  | EU482831 | EU687195 | EU862833 | KC155254 |
|  | EU482832 | EU862823 | EU862834 | EU155378 |
|  | EU482873 | EU862824 | JQ914271 | EU155379 |
|  | EU155380 |  |  |  |
|  |  |  |  |  |
| Japan | D10749 |  |  |  |
|  |  |  |  |  |
| Switzerland | EU255927 | EU255956 | EU255959 | EU256071 |
|  | EU255929 | EU255957 | EU256069 | EU256072 |
|  | EU255930 | EU255958 | EU256070 | EU862836 |
|  | EU255945 | EU255949 | EU255954 | EU255952 |
|  | EU255947 | EU255951 | EU255955 | EU255953 |
|  |  |  |  |  |
| Thailand | GQ913865 | HM042041 | HM042044 | HM042047 |
|  | GQ913867 | HM042042 | HM042045 | HM042048 |
|  | HM042038 | HM042043 | HM042046 | HM042049 |
|  | HM042039 | HM042040 |  |  |
|  |  |  |  |  |
| United Kingdom | AM600914 | AM600924 | AM600927 | AM600936 |
|  | AM600916 | AM600926 | AM600929 | AM600938 |
|  | AM600920 | AM600934 | AM600930 | AM600942 |
|  | AM600922 | AM600955 |  |  |
|  |  |  |  |  |
| United States | JX472013 | JX472005 | FJ958636 | FJ958700 |
|  | KX621532 | JX472007 | FJ958634 | FJ959020 |
|  | FJ896342 | FJ896309 | FJ958648 | FJ959027 |
|  | EU239713 | JX463563 | FJ958785 | KX621543 |
|  | EU255998 | JX463558 | FJ958791 | KX621526 |
|  | EU155286 | FJ896326 | FJ958784 | JX112543 |
|  | EU256019 | EU256040 | FJ958380 | JX112550 |
|  | FJ896357 | EU260396 | FJ958499 | JX112576 |
|  | FJ896366 | EU155239 | FJ958885 | JX112590 |
|  | KX621536 | EU256014 | FJ958882 | KP668763 |
|  | FJ896292 | EU256039 | FJ958707 | KP668776 |
|  | JX463556 | EU155310 | FJ896327 | KY189861 |
|  | EU482834 | KP668727 | JX463540 | KY189856 |
|  | EU529679 | KP668717 | KP668733 | NC004102 |
|  | EU255999 | EU256032 | KP668720 | EU482871 |
|  | EU155294 | EU482865 | FJ896340 | EU155297 |
|  | EU255997 | EU255938 |  |  |

**Supplementary Material Table S3.** Amino acids substitutions in NS5A protein not previously associated with resistance to protease inhibitors.

___________________________________________________________________

Genotype 1a strains Residue number Wild type Variant *n*

_____________________________________________________

36 F L 11

37 V I 1

37 V M 1

37 V L 1

44 R K 7

46 V T 1

46 V A 1

48 R K 3

50 D E 1

52 I V 1

64 T A 2

64 T S 1

68 K R 1

71 T S 1

73 R K 1

78 R/G K 11

78 R/G R 9

81 R K 1

85 S N 1

90 M/I I 19

105 N S 1

107 K/T K 9

107 K/T I 1

107 K/T E 3

107 K/T T 6

114 S A 1

117 E D 1

123 Q/R Q 13

123 Q/R R 7

126 D E 1

131 T S 1

133 M V 5

135 T/A T 15

135 T/A A 5

137 N D 3

144 V I 1

152 E Q 1

153 L V 1

153 L I 1

158 L I 1

**Supplementary Material Table S3.** Amino acids substitutions in NS5A protein not previously associated with resistance to protease inhibitors. (Cont.)

_____________________________________________________________________

Genotype 1a strains Residue number Wild type Variant *n*

__________________________________________________________

161 F Y 2

171 D/E E 14

171 D/E D 6

174 S T 2

176 R K 2

176 R V 4

181 D/E E 10

181 D/E D 8

181 D/E A 1

181 D/E V 1

183 P L 3

197 A S 1

211 A V 1

213 A T 6

215 G K 4

215 G R 3

219 A T 1

219 A E 1

226 V M 4

226 V L 10

237 P A 1

241 A G 4

245 T/A A 6

245 T/A T 12

245 T/A H 1

246 N K 1

247 H Y 1

252 A T 1

255 I L 2

256 E A 1

256 E Q 3

258 N S 1

264 E A 4

266 G K 1

266 G N 1

267 G D 1

267 G S 1

268 N D 1

269 M L 1

272 N A 1

**Supplementary Material Table S3.** Amino acids substitutions in NS5A protein not previously associated with resistance to protease inhibitors. (Cont.)

__________________________________________________________________

Genotype 1a strains Residue number Wild type Variant *n*

_________________________________________________________

275 E K 1

276 V I 1

280 I V 5

285 D E 4

288 V A 1

289 A T 1

290 E D 1

293 E G 1

294 E K 1

296 I/V I 11

296 I/V V 8

298 V T 1

299 P A 1

305 K R 4

308 R K 4

310 T/A T 8

310 T/A A 9

310 T/A G 1

311 P A 1

311 P Q 4

315 I V 8

326 L V 2

330 K R 4

331 K R 2

332 E K 1

335 E N 1

343 P P/R 1

348 Q R 6

349 S T 1

352 V I 1

_____________________________________________________________________

Genotype 1b strains Residue number Wild type Variant *n*

__________________________________________________________

34 V I 5

37 L/F L 7

37 L/F F 3

37 L/F I 1

44 K R 3

48 R H 1

49 G V 1

53 M V 1

54 Q/H Q 2

54 Q/H H/L 1

54 Q/H H 8

64 T P 2

65 G D/G 1

75 V A 1

78 K Q 1

78 K R 6

79 T S 1

83 T M 1

101 S T 3

107 S T 1

108 R K 1

108 R T 1

114 A A/T 1

122 T V 1

126 D E 1

130 V I/V 1

130 V I 1

135 T A 4

138 V I 2

138 V L 3

143 Q Q/L 1

145 P P/S 1

153 V L 2

158 L/F L 10

160 R R/S 1

164 A T 1

164 A S 1

171 E/D E 4

171 E/D D 7

173 V T 3

174 T S 1

174 T V 1

176 Q L 2

181 Q H 1

182 Y F 1

183 L P 3

183 L V 3

187 Q Q/L 1

196 V A 1

197 T/A A 1

197 T/A S 2

197 T/A T 8

198 V I 1

199 L V 2

205 D D/V 1

208 H Y 1

213 T A 2

215 K R 1

218 L M 1

226 L V 1

232 S S/C 1

232 S T 1

236 A A/E 1

236 A T 1

237 P L 1

**Supplementary Material Table S3.** Amino acids substitutions in NS5A protein not previously associated with resistance to protease inhibitors. (Cont.).

_____________________________________________________________________

Genotype 1b strains Residue number Wild type Variant *n*

__________________________________________________________

239 L M 1

240 K R 1

241 A A/T 1

242 T A 1

244 T A 1

245 T A 1

245 T G 1

246 H/R C 2

246 H/R H 5

246 H/R R 3

246 H/R C/Y 1

252 A I 1

255 I V 2

260 L M 1

262 R H 1

268 N D 1

269 I L 1

270 T I 1

275 E K 1

276 N K 1

280 I V 1

283 S P 1

285 E/D E 1

285 E/D D 9

285 E/D V 1

288 R Q 1

289 A M 1

291 E G 1

292 D G 1

292 D D/Y 1

294 R G 2

296 V I 2

298 V I 3

299 P A 1

305 K R 1

306 S T 1

307 R K 1

308 K N 1

311 P S 3

311 P A 1

313 M L 8

315 I V 5

326 I/L L 10

326 I/L V 1

328 S P 4

330 K K/R 1

**Supplementary Material Table S3.** Amino acids substitutions in NS5A protein not previously associated with resistance to protease inhibitors. (Cont.).

_____________________________________________________________________

Genotype 1b strains Residue number Wild type Variant *n*

__________________________________________________________

330 K R 3

331 D N 1

335 V T 1

336 P P/H 1

338 V L 1

339 V V/A 1

347 T A 2

349 A T 2

352 I V 4

NS5A protein amino acid substitutions found in HCV genotype 1a (n=20) and genotype 1b (n=11) patients are shown. *N* denotes the number of isolates harbouring the indicated substitution.

**Supplementary Material Table S4.** Amino acids substitutions in NS5B protein not previously associated with resistance to protease inhibitors.

___________________________________________________________________

Genotype 1a strains Residue number Wild type Variant *n*

231 S N 2

235 T V 1

238 A S 1

246 D A 1

248 Q E 1

251 V Q 1

251 V I 1

251 V M/V 1

252 A A/V 1

262 V I 1

269 S S/Stop 1

270 R K 3

272 E Q 1

276 T Y 23

296 Y F 1

297 I L 1

300 Q K 1

300 Q S 1

300 Q R 5

307 G K 1

308 L L/I 1

309 Q/R R 14

309 Q/R Q 11

311 C I 1

312 T K/R 1

327 A E 1

327 A Q 6

329 V I 1

330 Q R 1

335 S S/G 1

335 S N 3

337 R G 1

338 A V 1

365 S L 1

377 A A/T 1

377 A T 1

377 A V 1

382 T Y 16

415 F Y 1

421 A V 1

425 L M 1

426 M M/I 1

434 I L 11

451 C R 2

452 Y R 2

461 P Q 1

473 S T 1

487 A T 1

501 R R/S 1

506 S N 4

510 K/R R 8

510 K/R K 5

**Supplementary Material Table S4.** Amino acids substitutions in NS5B protein not previously associated with resistance to protease inhibitors (Cont.).

_____________________________________________________________________

Genotype 1b strains Residue number Wild type Variant *n*

231 N/S N 8

231 N/S S 3

242 A V 1

249 A V 1

252 A V 1

270 K L/Stop 1

273 N C 1

293 L I 1

300 S T 1

300 S A 1

309 Q R 1

316 C/Y C 11

329 T I 1

335 S N 3

355 Q R 2

374 H Y 1

393 A S 1

401 R K 3

403 T T/I 1

405 V I 1

421 A V 2

424 I V 1

426 M L 2

432 I V 1

434 L Q 1

437 E D 1

440 E G 1

444 D E 2

451 C Y 1

452 Y H 1

464 Q E 4

487 S A 1

499 V A 3

514 Q R 1

515 G E 1

NS5B protein amino acid substitutions found in HCV genotype 1a (n=20) and genotype 1b (n=11) patients are shown. *N* denotes the number of isolates harbouring the indicated substitution.
